# Characterization of Artifacts Produced by Gel Displacement on Non-invasive Brain-Machine Interfaces during Ambulation

**DOI:** 10.3389/fnins.2016.00060

**Published:** 2016-02-25

**Authors:** Álvaro Costa, Rocio Salazar-Varas, Andrés Úbeda, José M. Azorín

**Affiliations:** ^1^Brain-Machine Interface Systems Lab, Systems Engineering and Automation Department, Miguel Hernández UniversityElche, Spain; ^2^Center of Research and Advanced Studies (Cinvestav)Monterrey, Mexico

**Keywords:** artifact, human gait, brain-machine interface, conductive gel, electroencephalography, rehabilitation

## Abstract

So far, Brain-Machine Interfaces (BMIs) have been mainly used to study brain potentials during movement-free conditions. Recently, due to the emerging concern of improving rehabilitation therapies, these systems are also being used during gait experiments. Under this new condition, the evaluation of motion artifacts has become a critical point to assure the validity of the results obtained. Due to the high signal to noise ratio provided, the use of wet electrodes is a widely accepted technic to acquire electroencephalographic (EEG signals). To perform these recordings it is necessary to apply a conductive gel between the scalp and the electrodes. This work is focused on the study of gel displacements produced during ambulation and how they affect the amplitude of EEG signals. Data recorded during three ambulation conditions (gait training) and one movement-free condition (BMI motor imagery task) are compared to perform this study. Two phenomenons, manifested as unusual increases of the signals' amplitude, have been identified and characterized during this work. Results suggest that they are caused by abrupt changes on the conductivity between the electrode and the scalp due to gel displacement produced during ambulation and head movements. These artifacts significantly increase the Power Spectral Density (PSD) of EEG recordings at all frequencies from 5 to 90 Hz, corresponding to the main bandwidth of electrocortical potentials. They should be taken into consideration before performing EEG recordings in order to asses the correct gel allocation and to avoid the use of electrodes on certain scalp areas depending on the experimental conditions.

## 1. Introduction

Non-invasive Brain-Machine Interface (BMI) technologies have become very popular during the last decade. These technologies are based on the measurement of brainwaves using electrodes located over the scalp. Since it is based on passive sensors, this placement does not require surgery and has no other medical implications. By analysing brain potentials it is possible to decode several parameters related to the mental state of the subject. Biologically, this process is mainly performed (in a complex way) in the cerebral cortex, in the cerebellum and in the basal ganglia (Cisek and Kalaska, 2010; Mirabella, 2014). Normally motor commands are sent to the peripheral nerves and muscles through the spinal cord. However, in Spinal Cord Injured (SCI) patients, these communication pathways are interrupted at different levels depending on the severity of the injury. BMIs provide an alternative way to communicate the brain with external devices or even, indirectly, with the rest of the body using exoskeletons. In Carlson and Millán (2013) a BMI is used to control a wheelchair. In Escolano et al. (2012) a telepresence mobile robot is BMI-controlled allowing handicapped people to perform daily tasks. Currently, many works based on this technology have been oriented to rehabilitation. Classic rehabilitation techniques are based on therapist-patient interaction (O'Sullivan et al., 2013). Recent techniques are trying to obtain more natural movement patterns introducing exoskeletons in the rehabilitation process (Chen et al., 2013; Metzger et al., 2014). As these emerging systems are controllable devices, BMIs have been proposed by several researchers (Daly and Wolpaw, 2008; Pfurtscheller et al., 2008; King et al., 2013) as a method for increasing the level of the patients' involvement in the rehabilitation process. In the last 30 years it has been demonstrated that, both in humans (e.g., Elbert et al., 1995) and animal models (e.g., Lebedev et al., 2000), a wide range of experiences promotes physical changes of the brain structure. This brain plasticity represents the neural basis of learning and memory (for a review see Kolb and Gibb, 2014). It has been hypothesized that neuroplasticity could help functional recovery following brain injury by maximizing the use of the spared circuitry. In fact, some works have shown an increase in plastic changes in patients when they experience higher levels of involvement on their rehabilitations (Dimyan and Cohen, 2011; Kaneko et al., 2014). In Ang et al. (2014), a BMI based on motor imagery tasks is used to control a Haptic Knob robot providing patients a way to be involved with their rehabilitation.

Notwithstanding the remarkable results achieved with BMIs (Nicolelis and Lebedev, 2009), this approach still have limitations which explain why we are still far from approximating voluntary behavior using external devices. For instance, the speed of information transfer rates is currently not high enough (Baranauskas, 2014). Another problem is that the current understanding of brain processes underlying motor decision-making is not accurate enough. For example, volitional inhibition or the ability to cancel pending actions (Logan, 1994) has been disregarded several times by the BCI scientific community (Mirabella, 2012). Although there have been attempts to face this issue (Ifft et al., 2012), the current understanding of volitional control is not enough to properly adapt behavior to unattended changes either in the external environment or in our thoughts. In addition in the last few years, BMIs have been applied on lower limb rehabilitation. These studies involve the acquisition of data during gait processes and other body movements produced during ambulation (Chéron et al., 2012; Duvinage et al., 2012). The use of BMIs on these conditions involve several issues that should be addressed. The volume conduction of the scalp and the consequent smearing of signals produce a poor signal spatial resolution (da Silva, 2004) during electroencephalografic (EEG) acquisition. This scalp property produces the appearance of redundant information on nearby scalp areas. Due to volume conduction, several signals from other sources (artifacts) can be coupled to the EEG signals of interest. Usually, BMI research is performed on movement-free conditions where only electro-ocular and facial artifacts (Fatourechi et al., 2007) need to be taken into account. However, when a subject is walking, artifacts must be properly studied to know if there are new sources of noise affecting the recorded signals. To understand the importance of this study it is necessary to have a global view of the possible noises affecting EEG signals and knowledge of the state of the art techniques or methods developed to detect, reduce and removal of such artifacts. Depending on their source, EEG artifacts can be divided into three large groups: biological artifacts, environment artifacts, and equipment artifacts.

Biological artifacts are signals from biological sources that distort the EEG activity. Some of these signals come from physiological sources like blood pressure or skin tension. They have low and constant influence on EEG signals so the usual way of correcting them is by using a reference electrode. Others have an electrical source like electrooculographic (EOG) signals, electromyographic (EMG) signals or even other EEG potentials that mask the signals of interest. The appearance of these artifacts can lead to misleading results and to the development of systems that are really not studying the targeted phenomena. For that reason, there are lots of studies focused on understanding the nature of these signals in order to learn how to detect and reduce them. There are studies characterizing the influence of EMG signals and the scalp areas affected by them (Goncharova et al., 2003). There are several methods oriented to reduce EOG artifacts, from their removal by visual inspection to the use of linear regression techniques (Schlögl et al., 2007). More complex studies use independent component analysis (ICA) to decompose the signals into independent components and remove those related to artifacts (Gwin et al., 2010; Akhtar et al., 2012).

Environment artifacts are produced by the surrounding conditions of the experimental environment. Loud noises, flashlights and other visual stimuli can produce the appearance of evoked EEG potentials (Mitzdorf, 1985) that contaminate the EEG phenomena under research. Other factors like floor vibrations or electromagnetic fields produced by external devices can affect the subject and the equipment devices adding undesired signals coupled to the recorded data. These artifacts can be avoided by performing the experiment in a controlled isolated environment.

Finally, equipment artifacts are produced by those devices and methods included in the experiment. The most common is the power line interference which is a 50/60 Hz (depending on the world region) signal affecting the electrical network that gets coupled with the recorded data. Current systems include a 50/60 Hz notch filter to remove this interference. It can also be removed by connecting all the equipment to an isolation transformer. Most of these artifacts depend on the specific conditions of each experiment and they should be characterized to develop a systematic protocol that contribute to their removal or reduction. Also, current studies (Castermans et al., 2014; Kline et al., 2015) have found motion artifacts focalized on low-delta and high-gamma bands on the EEG signals during ambulation and head movements. In non-invasive BMI systems based on wet electrodes there is a common source of noise shared by many experiments. This technology is based on the placement of electrodes over the scalp applying a conductive gel between both surfaces. To easily place the electrodes over the scalp, an elastic cap that fits the subject's head is used. When an experiment does not require head movements, the conductive gel and the electrodes do not experience changes by settlement. However, when the experiment is performed during ambulation, due to the elasticity of the cap, the electrodes and the gel present displacements that induce undesired changes of the signals' amplitude. Both electrode and gel are not fully fixed elements of the acquisition system, electrode movements induce gel displacement and viceversa. As a consequence, the conductivity between the scalp and the electrode change during these movements producing changes in the signals' amplitude. For simplification reasons, hereafter, conductivity changes are going to be referred as gel movements as they could be seen like relative changes of position between the three elements that conform the acquisition system: scalp, gel and electrode. These artifacts have been considered on other works as artifacts from unknown sources. To remove them, data rejection techniques are usually used producing, in some cases, a significant decrease in the amount of data available for each study. This problem has become the main motivation to evaluate and understand these artifacts. Having these artifacts characterized, makes possible the development of experimental protocols focused on their avoidance.

The main goal of this work is to characterize, during EEG recordings, two types of noise produced by the displacement of conductive gel comparing ambulation vs. non-ambulation (thereafter referred to as movement free) conditions. These artifacts can be easily identified by measuring the electrodes' impedance, unfortunately, not all acquisition devices allow the computation of this parameter. To obtain the optimal signal, both noises should be evaluated. The first noise identified is produced by an initial misplacement of gel, producing a significant increase in the signals' amplitude and standard deviation over a whole run. The second noise is manifested as a sudden amplitude increase in a long segment of one run. To perform this study, EEG data from four experiments (three performed during ambulation and one performed movement-free) are used. To understand the nature of these artifacts, noisy channels are identified after evaluating parameters related to the signal's amplitude. Noisy channels are isolated and divided into groups depending on the type of noise, the experimental conditions and the scalp areas evaluated. The characterization of these noises is necessary to properly understand how they affect the EEG recordings and to develop experimental protocols oriented to avoid them in experiments involving human gait. To our knowledge this is the first study to carry out the characterization of this artifacts. Current works usually discard noisy electrodes and signal trials affected by these artifacts to perform their analysis. This could be a valid approach for offline analysis but not valid to perform online studies. In addition, the amount of information loss after applying data rejection techniques from gait experiments is a factor to take into account. If the scalp areas affected by these artifacts are known, it is possible to avoid them during experiments. It will be also possible to develop gel allocation protocols that reduce their influence. This approach is also helpful for avoiding unnecessary trial rejections and the consequent loss of information.

## 2. Materials and methods

### 2.1. Experiments and data sets

To perform this study, EEG data from four experiments are analyzed. Data sets 1, 2, and 3 are registers from experiments performed under the framework of BioMot (Smart Wearable Robots with Bioinspired Sensory-Motor Skills), an European project oriented to the development of an exoskeleton controlled by physiological signals for lower limb rehabilitation. These experiments were oriented to evaluate several parameters associated to the human gait. In them, EEG signals were recorded during treadmill walk. On the other hand, data set 4 is composed of EEG data related to motor imagery tasks where participants were not moving. Below, each set of recordings is presented with the relevant information related to the experiments performance, the environmental conditions and the procedure.

**Figure 1:**
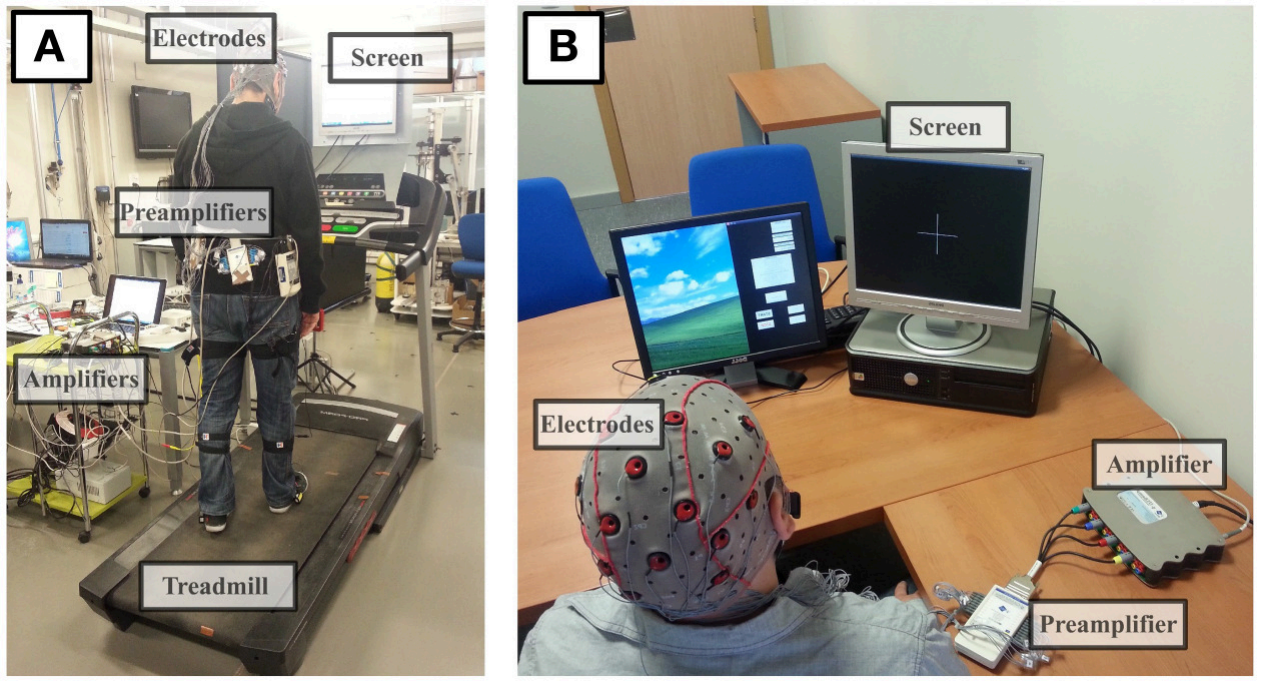
**Experimental conditions**. Panel **(A)** shows the experimental conditions of the experiment performed during ambulation: amplifiers are on the desk connected by cable to the preamplifiers. Electrodes are placed over the subject who is walking on a treadmill. A computer screen is placed in front of the subject to provide, if needed, visual feedback. All unnecessary electronic devices were disconnected during experiments and signals were visually analyzed prior to the experiment performance to confirm the absence of environmental noises affecting the recordings. Panel **(B)** shows the experimental conditions of motor imagery experiments: the subject sits in front of a screen that provides feedback about the experimental procedure. Signals are acquired using the electrodes placed over the scalp before the preamplification and digitalization stage.

The evaluated recordings have been acquired from two different experimental conditions. Figure 1A shows the experimental condition of data sets 1, 2, and 3. In this case, participants carried the equipment and walked on a treadmill. On the other hand, Figure 1B shows the second condition, where participants sat in front of a computer screen performing motor imagery tasks. Human data presented in this article have been acquired under an experimental protocol approved by the ethics committee for experimental research of the Miguel Hernández University of Elche, Spain. Written consent according to the Helsinki declaration was obtained from each participant. Table 1 shows specific participant and run features regarding each experiment. The specific conditions of the experiments were defined to fit the main goals of different studies. In the current study, these data sets are used to evaluate the observed artifacts produced by gel displacement.

**Table 1:** **Experiment specifications**.

	**Number of participants**					**Run duration**		
**Data set**	**Male**	**Female**	**Ages(mean ± STD)**	**Sessions**	**Runs per session**	**(min)**	**Total number of runs**	**Condition**
1	3	0	26.66 ± 4.04	1	8	5	24	Ambulation
2	8	2	26.60 ± 3.94	2	8	4	160	Ambulation
3	3	0	25.66 ± 2.88	1	16	3	48	Ambulation
4	12	0	27.33 ± 4.67	1	12	4	144	Movement-Free

Specific values of each data set regarding the number of participants, age, number of sessions, runs, and duration. All subjects were right-handed.

#### 2.1.1. Data set 1 (speed and tilt changes during gait)

The participants were asked to change several times their speed and tilt level. The sequence of tasks was: walk at 2 km/h and 10° of tilt, walk at 2 km/h and 5° of tilt, walk at 2 km/h and 0° of tilt, walk at 3 km/h and 0° of tilt, and walk 4 km/h and 0° of tilt. Each condition was performed for 60 s. Tilts positive values refer to positive inclinations. During a whole run participants were always looking forward.

#### 2.1.2. Data set 2 (gait attention changes)

Participants were asked to walk over the treadmill at a constant velocity of 2 km/h and 0° of tilt. During a run, the participant performed four activities: normal walk looking to a white screen, walking while performing mathematical operations shown on a screen, walking while watching a video, and walking following some adhesive marks on the treadmill. The duration of each activity was fixed by the experimenter to 60 s periods each. In the last activity the adhesive marks were placed to provide an irregular gait cycle, so the participant had to look down to follow them.

#### 2.1.3. Data set 3 (obstacle appearance during gait)

During this experiment the participants walked while several visual stimuli were presented simulating the appearance of unexpected obstacles. The walking was performed at 2 km/h with 0° of tilt. Each run was composed of two different tasks. In the first task, the participants were asked to stop their gait for a second when a laser projection appeared over the treadmill. In the second one, they were asked to stop their gait for a second when they saw a change of the screen color. Each condition lasts for 90 s. During the laser projection task, participants were looking down to see the laser appearance.

#### 2.1.4. Data set 4 (motor imagery task)

The participants were asked to sit in front of a screen and imagine the performance of different motor tasks. In each run the participant were instructed to imagine four specific motor movements related to right and left limbs. During the whole experiment participants remained seated in a movement-free condition.

### 2.2. Data acquisition

**Figure 2:**
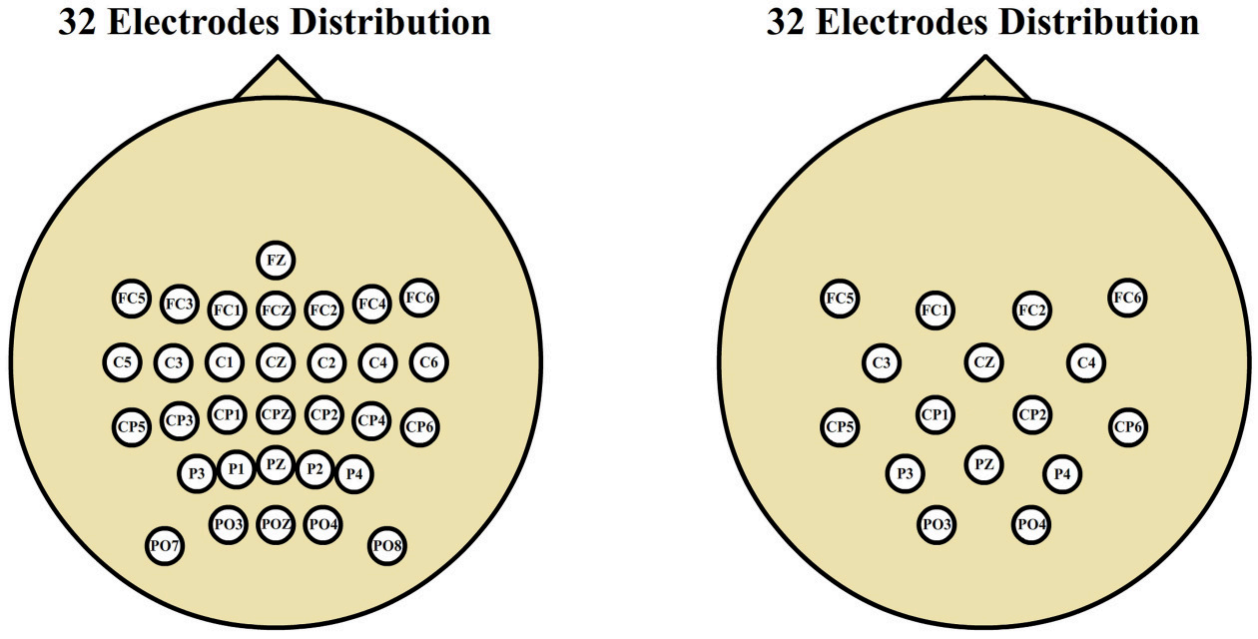
**Electrode distributions**. Thirty-two electrode scalp distribution corresponding to gait experiments and 16 electrode scalp distribution corresponding to motor imagery experiments. Both distributions cover the same spatial area with different spatial resolutions.

Data acquisition conditions were similar over all the data sets. The only appreciable changes were in the number of electrodes used. Thirty-two electrodes were used on ambulation recordings (data sets 1, 2, and 3) and 16 electrodes were used during movement-free recodings (data set 4). The spatial distribution of the electrodes was the same for all data sets, being smaller the spatial resolution on data set 4, as shown in Figure 2.

The EEG data were acquired using 32/16 pseudo-active electrodes to improve the signal to noise ratio with the distributions shown in Figure 2 according to the International System 10/10 (Klem et al., 1999) with a monoauricular reference in the right earlobe using AFZ electrode as ground. The scalp is measured to place the electrodes on the same anatomic areas for all participants (Towle et al., 1993). The conductive gel used to reduce the impedance between the electrodes and the scalp is a salt-base electrolyte gel (SignaGel, Parker Laboratories, USA). The electrical signals were preamplified (g.GAMMAbox, g.Tec, GmbH, Austria) and digitalized at 1200 Hz using two commercial amplifiers (g.USBamp, g.Tec, GmbH, Austria). These devices were also configured to apply a hardware low pass filter from 0.5 to 100 Hz, and a 50 Hz notch filter to remove the power line interference.

### 2.3. Analysis procedure

**Figure 3:**
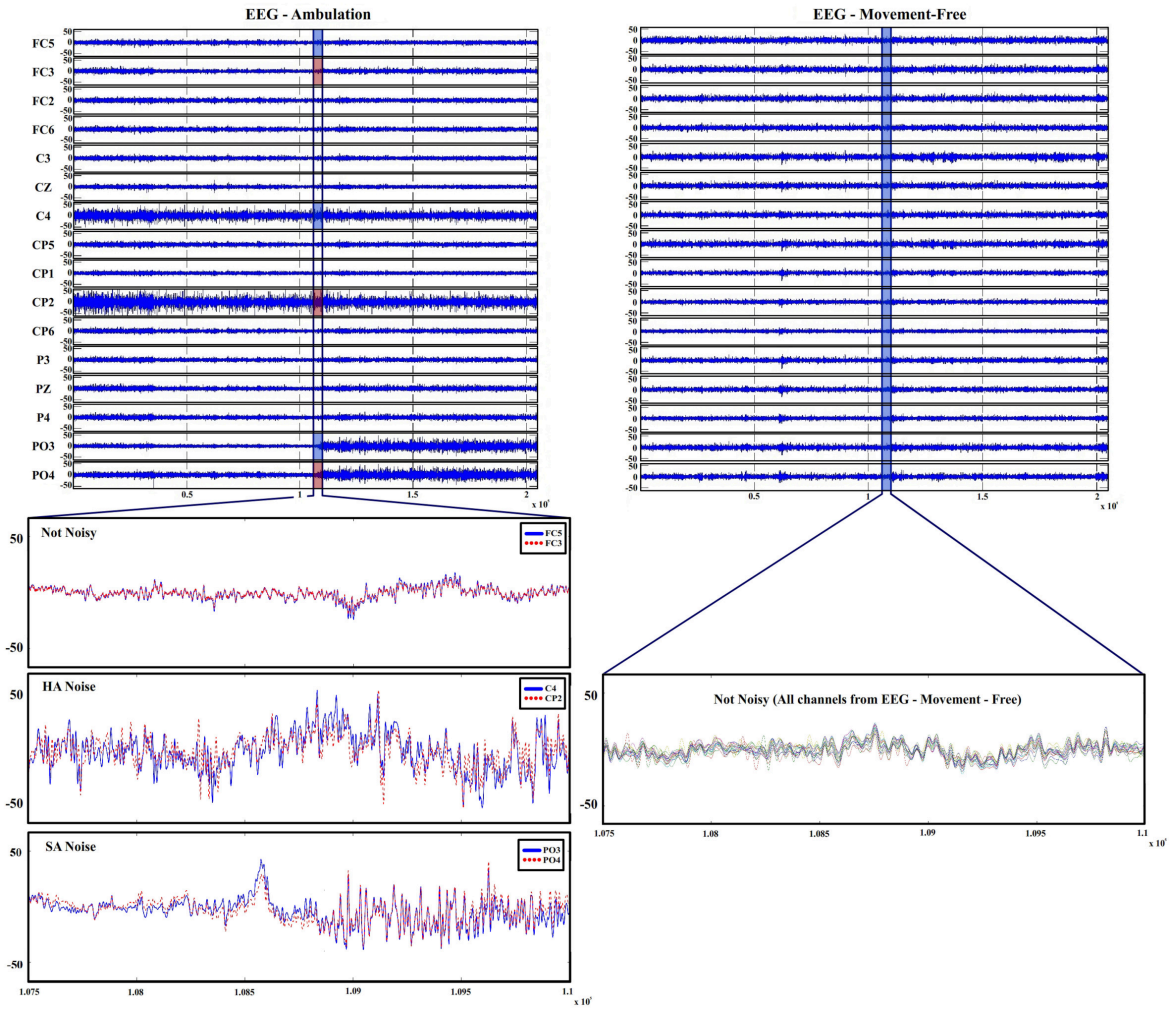
**EEG recordings during ambulation and movement-free conditions**. **Top left** shows EEG ambulation graphs with the data registered on channels from experiments performed during ambulation. **Bottom left** shows 2-s trials with detailed amplitudes and vibration of two not noisy channels (FC5 and FC3), two HA noise affected channels (C4 and CP2) and two SA noise affected channels (PO3 and PO4). **Top right** shows EEG movement-free graphs with the data registered on channels from the movement-free experiment. **Bottom right** shows 2-s trials with detailed amplitudes and vibration of all channels of EEG movement-free. Only the 16 electrodes shared on both distribution are shown to keep the figure dimensionality

The presented data sets were studied and compared throughout this section in order to characterize the equipment and set up artifacts produced on EEG signals during both conditions. In Figure 3, the 16 channels recorded from data set 4 (movement-free experiment) and the same 16 channels from data set 2 (ambulation experiment) are shown. By comparing both signals, two phenomenons mostly associated to ambulation data are described as follows:

1. **A sudden amplitude change** (hereafter, we call it as SA noise) as it is seen in electrodes FC5 and CP6.
2. **A higher amplitude** (hereafter, we call it as HA noise) during the whole run like those shown in C3 and CP5.

The run shown in Figure 3 has been selected for noise definition purposes. Not all the runs of ambulation data present both kind of noises and when they appear, they are not always associated to the same electrodes. Furthermore, the HA noise appears in some runs of data set 4 (movement-free). Regardless of their origin, all visible noise sources are associated to an unusual increase in the signal's amplitude. aAccording to literature, typical EEG amplitudes ranges differ throughout studies a(between 0.5 and 100 μV; Teplan, 2002 or between 50 and 200 μV; Srinivasan, 2007). It is important to characterize the typical amplitude of the recorded EEG signals in specific experimental and equipment conditions. In our case, it is clear that the increase of amplitude presented in electrodes FC5 and CP6, and the amplitude in C3 and CP5 are not produced just by EEG variations. In order to remove or reduce the appearance of these phenomena, it is necessary to know their origin. For that purpose, in this paper, both phenomenons are going to be studied using the four data sets previously introduced. To study and compare the signals, three parameters related to the amplitude of the signals are described:

1. Standard Deviation that measures the amount of variation or dispersion of a signal.
2. Segments Maximum Average (SMA) which is a parameter developed for this work that obtains the average of the maximum values of the signal once it has been divided into smaller segments. This parameter is related to the averaged maximum amplitudes of the signal during a run. The value obtained is similar to the average of the signal's envelope. The method followed to obtain this value is explained in the following sections.
3. Power Spectral Density (PSD) which provide a measurement of the power distribution across each frequency component.

#### 2.3.1. Analysis of the standard deviation

**Figure 4:**
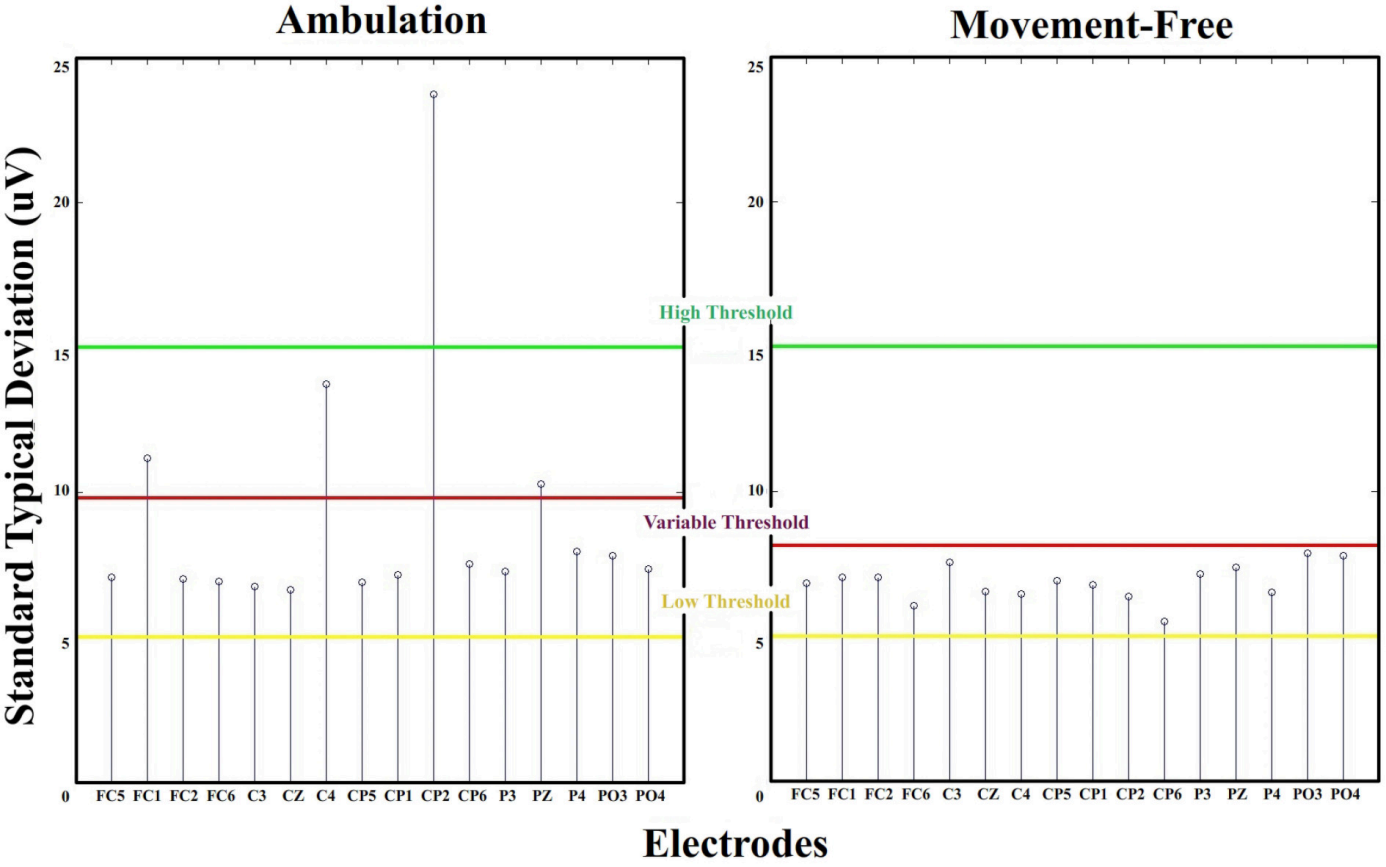
**Standard deviation thresholds**. Standard deviations for electrodes FC5 to PO4 from a run of ambulation and movement-free data. High threshold and low threshold are fixed. Electrodes above high threshold are classified as noisy, and electrodes under low threshold are classified as not noisy. For middle standard deviation values, the variable threshold is defined dynamically. Electrodes are classified noisy and not noisy depending on if they exceed or not this threshold.

The standard deviation of EEG signals is a widely used parameter due to the statistical properties of this waves. High pass filtered EEG signals follow a normal distribution with mean zero (Blanco et al., 1995). Also all channels are referred to a free of noise electrode consistently gripped to the right earlobe. Under this circumstances, the standard deviation can be considered constant for long trials. For that reason this parameter is commonly used for normalization purposes (Cincotti et al., 2008). Cortical signals recorded from different scalp areas have similar amplitudes and constant standard deviations. If a high amplitude noise appears in a channel during EEG recording, it can be easily detected by evaluating outliers standard deviation values from an electrode distribution. This method has been widely used in literature as data rejection technique (Gwin et al., 2010, 2011; Salazar-Varas et al., 2015). By studying the standard deviation of EEG recordings, it is possible to select the noisy electrodes in each run. Figure 4 shows the standard deviation values for each electrode of ambulation and movement-free runs from Figure 3. From this representation it is easy to distinguish the noisy electrodes. To perform an automatic labeling of the noisy electrodes of a run, two fixed thresholds and one variable threshold are applied to the values of standard deviation computed for each run (Figure 4):

- Variable Threshold: It is computed for each run as:
  (1) $$\begin{array}{l} Variable\;Threshold=SDAverage+\left(SDAverage-SDmin\right) \end{array}$$
  where *SDAverage* is the average value of the standard deviation of all the electrodes of one run, and *SDMin* is the minimum standard deviation value of all the electrodes of one run.
  Standard deviation values above this threshold are labeled as noisy electrodes and those under it are labeled as not noisy. This method performs correct classification during runs where just a few channels present noise contribution.
- High Threshold: It is fixed at 15 μ*V*. Standard deviation values above this threshold are classified as noisy. Its purpose is to avoid bad classification in the case that all electrodes present noise contribution. This is an uncommon scenario usually associated to abrupt conductivity changes on the ground electrode due to a bad allocation of the conductive gel.
- Low Threshold: It is fixed at 5 μ*V*. Standard deviation values under this threshold are classified as not noisy. Its purpose is to avoid bad classification in the case that all electrodes present not noise contribution. This is a common scenario where all channels show stable conductivity values between the scalp and the electrodes.

The range between both fixed thresholds (5 and 15 μ*V*) correspond to the typical values of standard deviations observed throughout runs of the 4 data sets. The variable threshold is defined to select noisy electrodes with standard deviation within this range.

In Figure 3, only electrodes FC5, C3, CP5, and CP6 from the ambulation data are labeled as noisy. This labeling is applied to each run for all data sets. The length of labeled vectors differs between data sets as they are recorded using different number of electrodes. Both kind of noises are recognized following this method. They share similar statistical parameters and the only difference between them is the moment where the amplitude changes. For that reason, once a channel is identified as noisy, it is classified as HA or SA by visual inspection. After performing this labeling over a set of runs and updating the value of the vector, it is possible to see which electrodes are more sensitive to the phenomena under research.

#### 2.3.2. Analysis of the segments maximum average (SMA) related with HA noise

**Figure 5:**
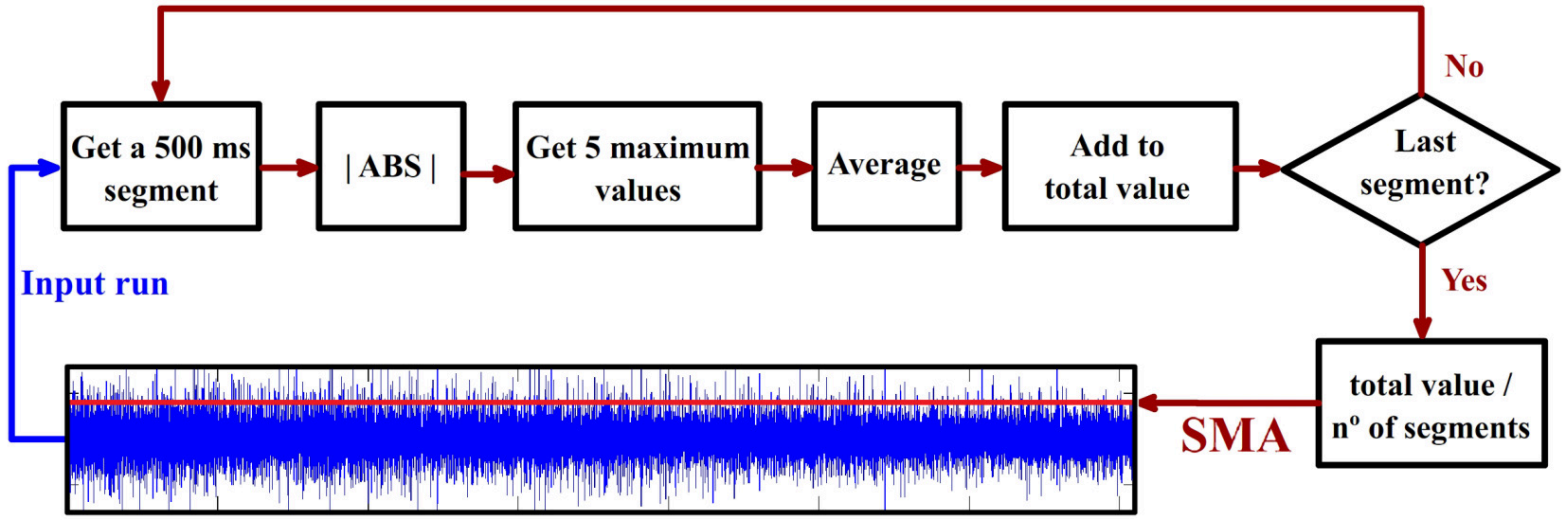
**Segments maximum average (SMA) calculus**. The time signal is divided into 500 ms consecutive non-overlapped epochs. The absolute value of the epoch is computed. The five maximum values for each epoch are averaged and the result is added to a variable called “total value.” This process is repeated on every epoch. The final value of “total value” is divided into the total number of epochs providing as a result the SMA parameter represented with a red line over the blue time signal. This value is calculated for all the electrodes and runs.

This parameter provides a measure about the average of the maximum values of the signal amplitudes once a run is divided into segments (the segmentation is trying to emulate real time conditions). On each run, the SMA is calculated for each channel. Figure 5 illustrates how this parameter is computed and the results provided in a random run and electrode. The length of the consecutive and non-overlapped segments used (500 ms) has been selected to fit the usual requirement of real time systems used in other works performed by our group (Hortal et al., 2015). This parameter is used to evaluate the evolution of the channel's amplitude across consecutive runs.

#### 2.3.3. Analysis of the noise power spectral distribution

The PSD of each run and electrode of all data sets is computed from 5 to 90 Hz with a spectral resolution of 1 Hz using the pwelch method (Welch, 1967). The output of this process provides 86 values vector per run and electrode (a total of 9728 frequency vectors counting all channels of all runs for the four data sets). Using the standard deviation previously described, these vectors are divided into two groups corresponding to noisy and not noisy signals. A Wilcoxon Sum-Rank test is performed with a confidence interval of 95% and applying a Bonferroni correction for multiple comparisons (Cabin and Mitchell, 2000) to validate the significance between noisy and not noisy signals at each frequency (86 statistical comparisons).

#### 2.3.4. Noise significance between ambulation and movement-free data

After dividing between HA and SA (see Section 2.2.1), noisy data are associated to the data set and channel where they were found. From this classification several parameters related to the distribution of noise are obtained: The number of HA noises (*HA*-*N*), the number of SA noises (*SA*-*N*), the total number of noises (*Total*-*N* = *HA*-*N* + *SA*-*N*) and the total number of channels evaluated (*Total*-*R* = *Total*-*N* + *Not noisy channels*). These parameters are used to compute the ratio of each noise against the total amount of noise (*HA*-*N* vs. *Total*-*N* and *SA*-*N* vs. *Total*-*N*) and against the total amount of channels analyzed (*HA*-*N* vs. *Total*-*R* and *SA*-*N* vs. *Total*-*R*). This ratios are computed for complete data sets (three values per ratio for ambulation data from data set 1–3 and one value per ratio for movement-free data from data set 4) and also for single channels of each data set (32 × 3 values per ratio for ambulation data and 16 × 1 values per ratio for movement-free data). These last vectors are used to test the significance in the apparition of the noises described between ambulation and movement-free data running a Wilcoxon Sum-Rank test (Wilcoxon et al., 1970) with a confidence interval of 95% and applying a Bonferroni correction for multiple comparisons.

## 3. Results

**Figure 6:**
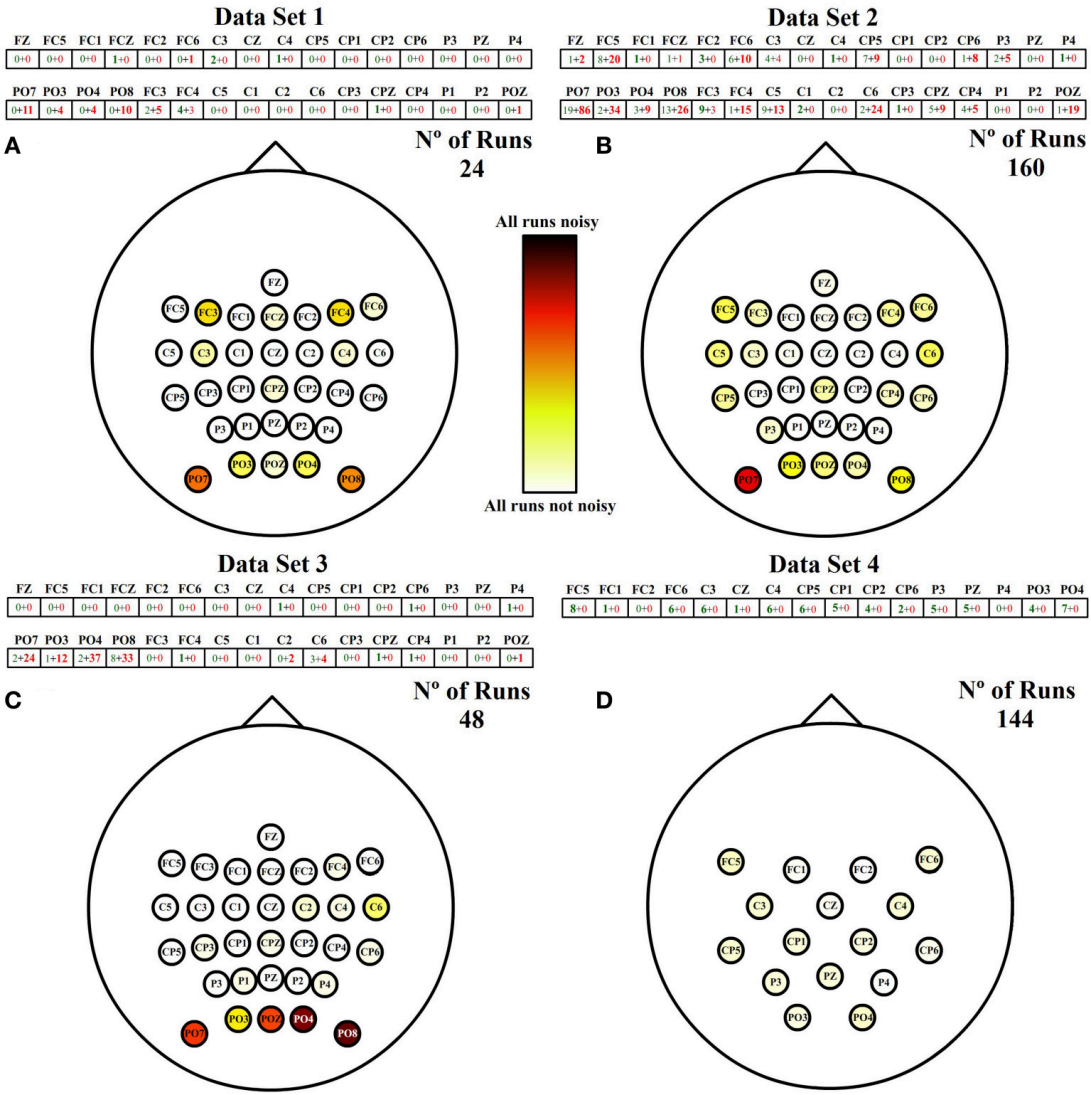
**Noises spatial distribution**. Each cell of the vector on the top of each scalp represents the number of times an electrode has been classified as noisy after the analysis of all runs. Each number is divided into two values. Green and red values correspond to the number of HA and SA noises identified, respectively. The total number of noisy runs per electrode are spatially represented over each scalp in red shade colors between white and black. **(A)** Spatial distribution of noisy channels for runs of data set 1. **(B)** Spatial distribution of noisy channels for runs of data set 2. **(C)** Spatial distribution of noisy channels for runs of data set 3. **(D)** Spatial distribution of noisy channels for runs of data set 4.

Figure 6 shows the labeled vector of each data set after applying the thresholds defined on the standard deviation of each run. Each vector is followed by a spatial representation of the electrodes in the scalp with different shades of red. The shades are assigned to each electrode depending on the following coefficient:

(2) $$\begin{array}{l} \frac{Number\;of\;noisy\;classi\mathrm{fi}ed\;runs}{Total\;number\;of\;runs\;analyzed} \end{array}$$

where the 0 is represented as white and 1 as black. This coefficient works as a standardization parameter to compensate the variable number of runs of each data set.

The addition of both numbers represented inside each electrode is the total number of noisy runs detected per electrode. The first factor (green) is the number of noisy runs produced by a high amplitude (HA) during the whole run, and the second factor (red) is the number of noisy electrodes produced by a sudden amplitude (SA) change. A deeper analysis of Figure 6 shows that data sets related to ambulation present higher numbers of noisy runs, being the noisiest electrodes those located on posterior peripheral scalp areas. In addition, ambulation data are principally contaminated by SA noises while movement-free data are only contaminated by HA noises.

**Figure 7:**
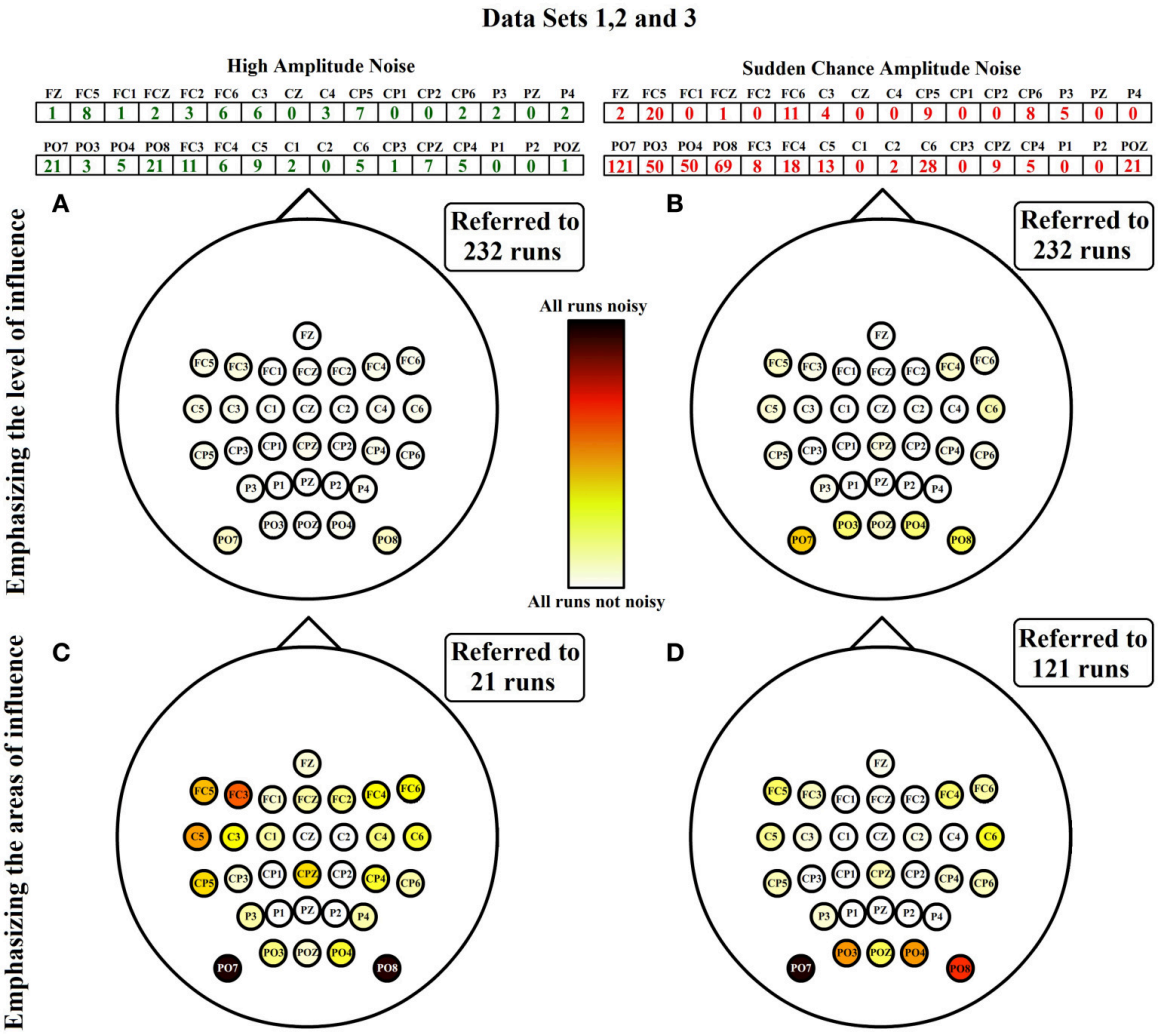
**HA and SA noises emphasizing level and areas of influence**. HA and SA noises from data sets 1, 2, and 3 are separately analyzed. On the first column, the sum of HA noises from data sets 1, 2, and 3 are represented. The same representation is shown on column two with SA noises. In the first row, noises are represented over the total number of runs analyzed, showing that way the level of influence of the noise along all the runs. In the second row, noises are represented over the maximum number in the classification vector, emphasizing that way the areas affected by each noise. **(A)** Spatial distribution of channels with HA noise referred to the total amount of runs evaluated. **(B)** Spatial distribution of channels with SA noise referred to the total amount of runs evaluated. **(C)** Spatial distribution of channels with HA noise referred to the maximum number of HA noisy channels identified. **(D)** Spatial distribution of channels with SA noise referred to the maximum number of SA noisy channels identified.

To appreciate the influence of each noise, Figure 7 shows the spatial distribution of both noises separately. For each noise there are two representations. The first one (Figures 7A,B) is referenced to the total number of runs of ambulation data (24 from data set 1 + 160 from data set 2 + 48 from data set 3 = 232 runs). This representation highlight the level of contamination that each noise produces in the data. The second one (Figures 7C,D) is referenced to the highest number of noisy runs (21 in the case of HA noise vector and 121 for SA noise vector) to emphasize the most affected areas of each noise. Figure 7 also shows how SA noise is very focused on peripheral areas, while HA noise affects most of the scalp with a lower ratio of occurrence. In the case of data set 4, all the noises detected are HA noises, suggesting that SA noises are caused by performing EEG recordings during ambulation. Table 2 shows the percentage of SA and HA noises against the total number of noisy electrodes and against the total number of channels in each data set. On ambulation experiments, HA noise represents the 21.49% of the noises and the 1.65% of the total amount of data. On the other hand, SA noises represents the 78.51% of the noises and the 6.11% of all the data evaluated. Moreover, on movement-free experiment, the only noise identified (100%) is the HA, representing the 2.86% of the total amount of data acquired under this condition. This table is useful to compare the HA noise results from movement-free data (Figure 6D) and the noise results from ambulation data (Figure 7A). In the last column, the confidence *p*-values provided by the Wilcoxon Sum-Rank Test, validate the significant differences between ambulation and movement-free experiments in terms of the comparisons performed.

**Table 2:** **SA and HA noises vs. total number of noisy channels and total number of channels analyzed**.

	**Ambulation**				**Movement-Free**	**Significance (*p*)**
	**Data set 1**	**Data set 2**	**Data set 3**	**Average**	**data set 4**	**Ambulation vs. Movement-Free**
HA-N (number of channels)	11	107	22	–	66	–
SA-N (number of channels)	39	302	113	–	0	–
Total-N (number of channels)	50	409	135	–	66	–
Total-R (number of channels)	768	5120	1536	–	2304	–
HA-N vs. Total-N (%)	22.00	26.16	16.30	21.49	100.00	**4.74·10^−59^**
SA-N vs. Total-N (%)	78.00	73.84	83.70	78.51	0.00	**4.74·10^−59^**
HA-N vs. Total-R (%)	1.43	2.09	1.43	1.65	2.86	**5.58·10^−8^**
SA-N vs. Total-R (%)	5.08	5.90	7.36	6.11	0.00	**3.28·10^−63^**
Total-N vs. Total-R (%)	6.51	7.99	8.79	7.76	2.86	**1.09·10^−35^**

SA-N and HA-N are the number of noisy channels affected by each kind of noise, Total-N is the sum of SA-N and HA-N and Total-R is the total number of channels analyzed. Each cell shows the relation between the parameters specified in the first column for each data set. Average values for all data sets related to the performed experiments during ambulation are also shown. In the last column, the confidence p-values provided by the Wilcoxon Sum-Rank Test, validate the significant differences between ambulation and movement-free experiments in terms of the comparisons performed.

**Figure 8:**
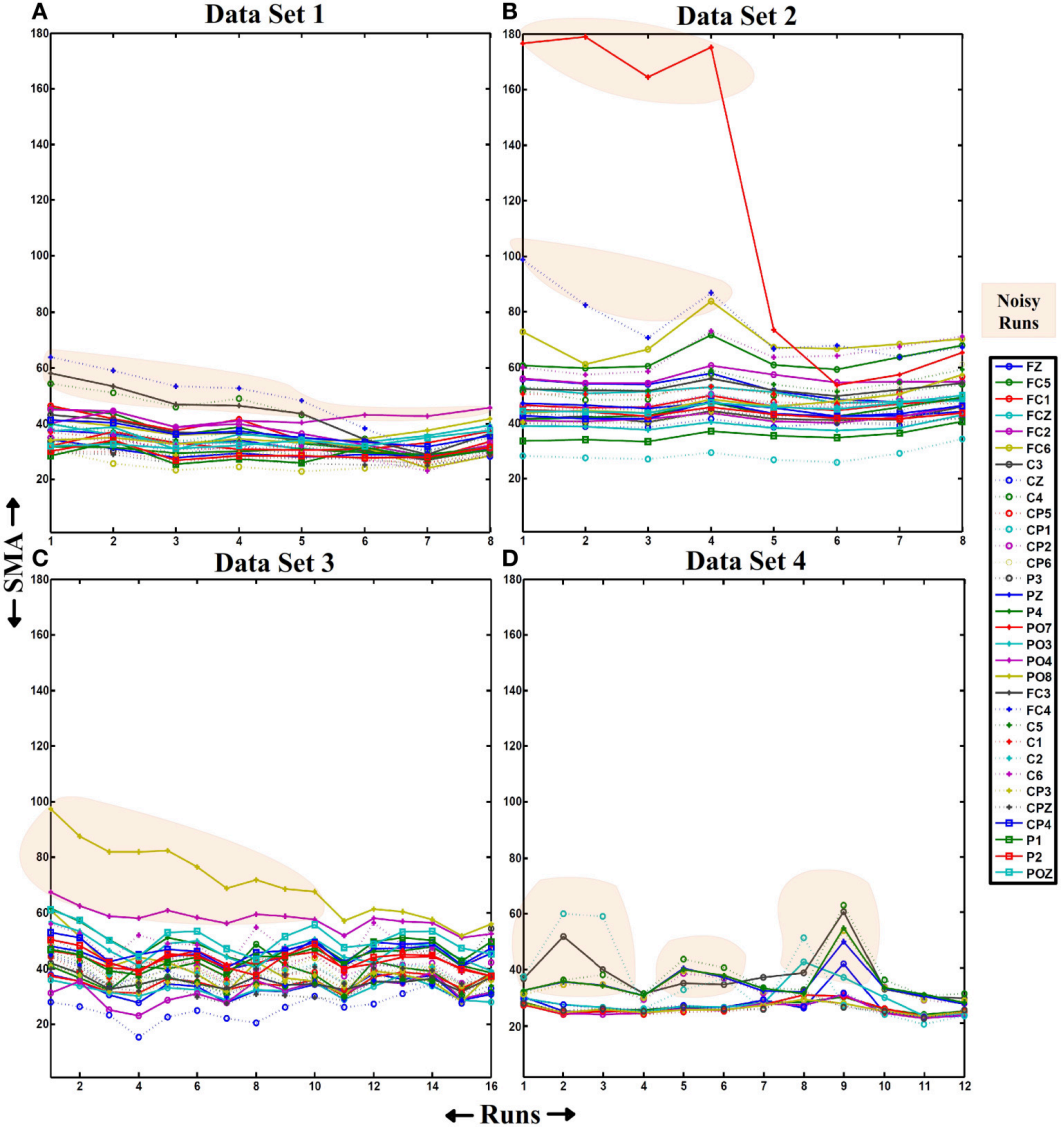
**SMA evolution**. On each graph, the X-axis represents the number of runs of a single session and the Y-axis represents the value of the SMA. This parameter is represented from all the runs of a session from data sets 1, 2, 3, and 4 (graphs **(A–D)**, respectively). The SMA values for all the electrodes and runs are represented. The number of runs on a session depends on the experiment/data set represented (8 runs for data sets 1 and 2, 16 runs for data set 3, and 12 runs for data set 4). Also, the number of channels is different (32 for data sets 1, 2, and 3 and 16 for data set 4). The orange shadow marks SMAs for electrodes and runs identified as noisy.

As shown in Figure 6, HA noise does not appear in a specific scalp area neither from ambulation nor from movement-free data. However, it has higher influence in movement-free data. To understand this phenomenon, the evolution of the amplitude of noisy electrodes is measured during a whole session. For each data set, a session where the HA noise phenomenon appears is selected and the SMAs of all electrodes are computed and represented for all the runs as shown in Figure 8. Each line corresponds to a EEG channel as shown in the legend. Runs of electrodes affected by HA noise (related to an unexpected high value of the SMA) are in the highlighted areas. Figures 8A–C corresponding to ambulation data (data sets 1–3) presents a similar behavior on their respective noisy electrodes. The noise decreases after the performance of several consecutive runs. On the other hand, Figure 8D, corresponding to movement-free data (data set 4), shows noisy electrodes presenting an erratic behavior during the whole session.

**Figure 9:**
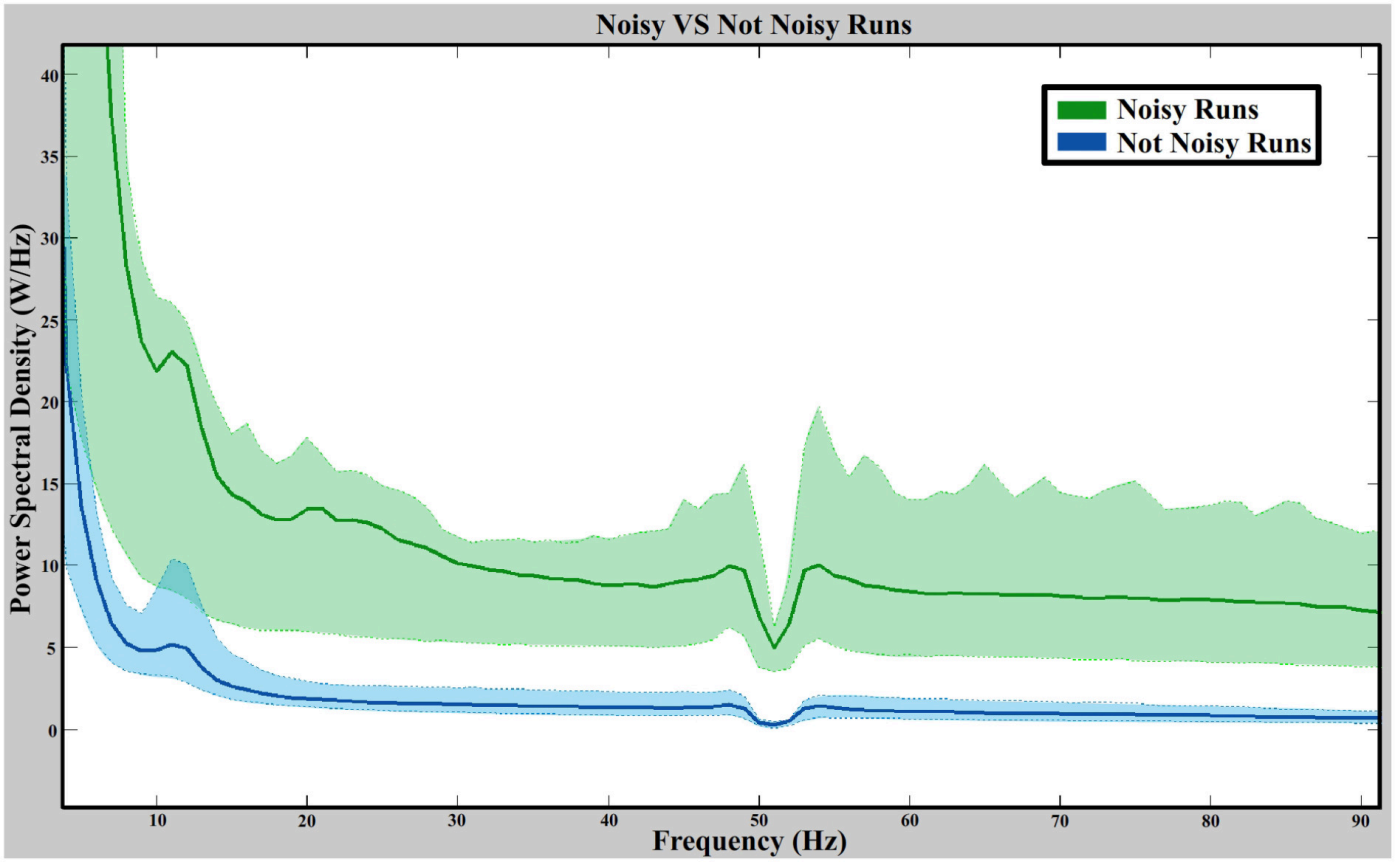
**Noisy vs. not noisy signals spectral distribution**. The green and blue straight lines are the average value of the noisy and not noisy signals (from all data sets), respectively. Green and blue dashed lines represent the 25 and 75% of each distribution. The power spectral distribution is computed from 5 to 90 Hz with a resolution of 1 Hz.

Figure 9 shows the average value of the spectral distribution of noisy signals (670 values per frequency corresponding to runs labeled as noisy) and not noisy signals (9058 values per frequency corresponding to runs labeled as not noisy). For each group, the 25 and 75% of each frequency is represented showing a clear PSD dominance of noisy signals over not noisy signals. The Wilcoxon Sum-Rank test is used to compare both groups (670 values for noisy signals and 9058 values for not noisy signals) at each frequency (86 frequencies). All confidence levels fulfill the confidence interval after applying a Bonferroni correction showing significance results between noisy and not noisy electrodes at all frequencies. The power decrease on 50 Hz is dued to the notch filter applied during the amplification stage.

## 4. Discussion

We identify two kind of noises (HA and SA) which are related to equipment artifacts. Interestingly, the main source of noises in EEG recordings performed during ambulation are SA noises appearing only during ambulation and representing the predominant type of noise presented in the EEG signal recorded. Moreover, this noise is focused on peripheral areas corresponding to those scalp locations more sensitive to conductivity changes during head reorientations. These findings are further supported after comparing the spatial distributions of the noises from data sets 1, 2, and 3 with the typical head movements from the three experiments performed during ambulation. The clearest example can be seen on experiment three. During this experiment participants were asked several times to keep a normal walk while they reoriented their heads to the ground in order to see the appearance of visual stimulus. This reorientation produced gel displacements on occipital areas of the scalp inducing the appearance of this noise. The experiment two has a close relationship with experiment three. In this case, participants are also asked to look down but this time in order to follow some marks (with a non-periodic step length) placed over the treadmill which produce an unsteady gait pattern with the consequent left and right head movements. These movements also provoked electrodes displacement over the lateral areas. Experiment one does not imply any specific head reorientation but it includes changes on the ambulation speed producing in this case a different pattern of head movement. Occipital areas are not so affected as participants do not look down. The appearance of noise on occipital and frontal areas was probably caused by the increase of the backward and forward movement of the head during fast ambulation.

On the other hand, HA noises appear in both ambulation and movement-free data. They represent, approximately, one fifth of the noises evaluated. In the case of movement-free data, they are the only kind of noise appearing in the signal. In this case, the HA noise has significant and greater effect in movement-free data than in ambulation data. In addition, this noise present low intensity affecting all scalp areas. These two facts support the hypothesized idea that this noise is produced by a bad gel allocation during the set up of the equipment. This error is committed in random electrodes when the control population is big and its effects are gradually reduced on each run of ambulation experiments. A possible explanation of these effects is that ambulation provokes gel settlement improving the conductivity between the scalp and the electrodes. During movement-free experiment, noisy electrodes present an erratic behavior during the full session suggesting that the gel was bad allocated during the first run and it did not settle along runs. This behavior is supported by the fact that participants kept their heads still during movement-free experiment.

Finally, the spectral power of the noisy trials shows significant values (at all frequencies) against not noisy trials. This suggest that the artifact measured are not directly related to the motion artifacts described on (Kline et al., 2015; affecting mainly low frequencies) nor to the delta and high-gamma band rhythms observed on (Castermans et al., 2014)

## 5. Conclusion

An analysis of set up artifacts produced by conductivity variations between the scalp and the electrodes has been performed. During this work, data from four different experiments have been used: three of them performed during ambulation, and the fourth one performed movement-free. Two different phenomena have been characterized.

An unusual increase in the signal amplitude of some electrodes (referred as HA noise in the paper) appears in both ambulation and movement-free data with a low apparition rate (1.68% in ambulation and 2.86% in movement-free) and a random scalp distribution. Results suggest that they are provoked by gel bad allocation during the electrode's set up. This unusual amplitude is presented in complete sessions from movement-free experiments. On the other hand, in ambulation experiments, the amplitude experiences a reduction throughout runs of the same session converging to typical amplitudes.

A sudden change in the signals' amplitude of some electrodes (referred as SA noise) appears only in ambulation data with a higher rate of apparition (21.49%) and focused on peripheral areas. Results suggest that they are provoked by gel displacements produced by head movements on areas of the scalp where the cap presents critical movements due to its elasticity. These areas change depending on the head movements performed. Looking down triggers this change on occipital areas, moving the head forward and backward on occipital and frontal areas, and right and left head movements affect lateral areas.

These noises can be easily distinguished from other physiological noises like EOG, EMG or cardiac rhythms. Contribution of EOG artifacts is only noticeable in a single trial basis (Elbert et al., 1985) and EMG due to arms and legs movements and cardiac rhythms do not change the amplitude of the signal as much as the artifacts described in this work (Freeman et al., 2003; Moretti et al., 2003). Only EMG signals produced by continuous jaw clenching or heavy head movements have similar effects in the EEG signals but, in this case, these effects hugely affect all the electrodes and not just a random set of them as it was found during this work.

So far, most of works related to BMI do not require the performance of movements. Most studies present artifacts produced by physiological factors, sometimes unavoidable during recordings, like blinks. To deal with them, there are methods like linear regression that allow their removal during real time analysis. Still, many studies apply data rejection techniques in offline analysis suggesting that there are equipment and set up artifacts affecting the electrodes during recordings. Data rejected is usually discarded. In these studies the amount of data rejected represents a small percentage of the total data recorded and it is mostly produced by the noises described in this work. With the appearance of a relatively new branch of research where EEG signals are measured during gait, the amount of equipment and set up artifacts significantly increases. In Gwin et al. (2011) the electrocortical activity during the gait cycle is studied, using 248 electrodes array to measure EEG signals during treadmill ambulation. After an electrode rejection technique, an average of 117.6 electrodes were discarded, meaning that the 47.41% of the recorded data was unused. The same data (again with this 47.41% of rejection) was used in Gwin et al. (2010) to implement a real time artifact removal during walking and running. This research is focused on the study of physiological artifacts (like electrical muscles activation) providing really useful findings and removal methods based on independent component analysis (ICA). Even so, a previous analysis like the one presented in this paper could help to reduce the number of rejected electrodes and to avoid the register of noisy areas of the scalp depending on the kind of movements performed. Future works that intend to acquire EEG signals during ambulation should take into consideration the problems addressed on this work to avoid the processing and storage complications coming from contaminated data.

As mentioned in the introduction, there are many potential noise sources during EEG recordings. This paper is focused on the study of a kind of noise produced by conductivity changes due to gel displacements which are, at the same time, specific equipment noises. This study is helpful to design efficient experiments but it is not enough to ensure the absence of artifacts from other sources. Many studies have proved the appearance of EMG contributions in EEG signals during muscle activation (Brown et al., 1999; Hansen and Nielsen, 2004). These physiological noises are usually not avoidable during recordings, however there are studies focussed on removing them with specific mathematical techniques (Schlögl et al., 2007; Gwin et al., 2010). Research oriented to evaluate EEG phenomena should take into account all kind of potential noise sources and deal with each one of them on the correct stage of the experimental procedure.

## Author contributions

AC and JA designed the study and contributed to writing the manuscript. AC analyzed dada and interpreted the results. All the authors participate conceiving the experiments. AC, RS, and AU collected the data and also participate in the drafting of the manuscript. All authors read and approved the final manuscript.

## Funding

This research has been carried out in the framework of the project BioMot - Smart Wearable Robots with Bioinspired Sensory-Motor Skills (Grant Agreement number IFP7-ICT-2013-10-611695), funded by the Commission of the European Union, and the project Associate - Decoding and stimulation of motor and sensory brain activity to support long term potentiation through Hebbian and paired associative stimulation during rehabilitation of gait (DPI2014-58431-C4-2-R), funded by the Spanish Ministry of Economy and Competitiveness and by the European Union through the European Regional Development Fund (ERDF) “A way to build Europe”.

### Conflict of interest statement

The authors declare that the research was conducted in the absence of any commercial or financial relationships that could be construed as a potential conflict of interest.
